# Preparation of oyster peptide and *Pfaffia glomerata* pressed candy and its ameliorative effect on sexual dysfunction in male mice

**DOI:** 10.1002/fsn3.4213

**Published:** 2024-05-13

**Authors:** Qianqian Huang, Haiying Wu, Xiangxin Xiao, Xiaoming Qin, Suqing Liu

**Affiliations:** ^1^ College of Food Science and Technology Guangdong Ocean University, Guangdong Provincial Key Laboratory of Aquatic Products Processing and Safety, Guangdong Provincial Science and Technology Innovation Center for Subtropical Fruit and Vegetable Processing Zhanjiang China; ^2^ National Research and Development Branch Center for Shellfish Processing Zhanjiang China; ^3^ College of Coastal Agricultural Sciences Guangdong Ocean University Zhanjiang China

**Keywords:** oyster peptide, paroxetine, *Pfaffia glomerata*, pressed candy, sexual dysfunction

## Abstract

Oyster peptide (OP) and *Pfaffia glomerata* extract (PGE) were used as raw materials. The optimal formulation of the pressed candy (PC) was optimized by one‐way experiment and D‐optimal mixture experiment design, and animal experiment was used to evaluate the effect of PC on male sexual dysfunction. The results showed that PC intervention significantly improved the sexual behavior of male mice with sexual dysfunction, including a significant shortening of the mount latency (ML) and intromission latency, and a significant increase in the mount frequency (MF) and intromission frequency (IF). At the same time, the concentrations of serum testosterone (T) and luteinizing hormone (LH) in mice were restored, and the erectile parameters and pathological changes of penile tissue were improved. Further studies found that PC intervention increased the activities of superoxide dismutase (SOD), catalase (CAT), and glutathione peroxidase (GSH‐Px) and reduced the content of malondialdehyde (MDA) in testicular tissue. In addition, PC intervention improved testicular tissue morphology. In conclusion, the obtained PC has good taste quality, and the relevant quality indicators are qualified. It has a good ameliorative effect on male sexual dysfunction and may be a potential dietary supplement.

## Graphical abstract

The purpose of this study is to use oyster peptide and *Pfaffia glomerata* extract as the main raw materials to develop a pressed candy (PC) with high nutritional value and for improving male reproductive dysfunction, so as to meet people's increasing demand for nutritious and healthy food, and promote the development of oyster peptide and *Pfaffia glomerata* deep processing industry, and provide a new way for the development of oyster peptide and *Pfaffia glomerata* products.

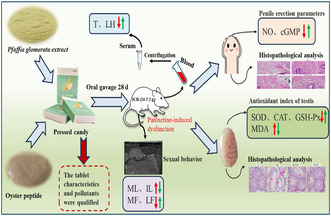

## 1 INTRODUCTION

Infertility is a global public health problem. About 8%–12% of couples in the world are affected by infertility, and male infertility accounts for 50% of all infertility cases (Kumar & Singh, 2022). Environmental factors, lifestyle and eating habits, and drug side effects are the main causes of male sexual dysfunction (Brannigan, 2019; Karavolos et al., 2020). Testis is the main organ for sperm production and androgen synthesis and secretion, and plays a key role in maintaining male sexual function. When testicular tissue is damaged, it may lead to difficulty in sperm synthesis and disorder of androgen secretion, thus affecting the normal fertility of men (Gao et al., 2023). The penis is the main organ for men to complete sexual life and semen discharge, therefore, male erectile dysfunction (ED) is also an important cause of male infertility (Zhou et al., 2021). Paroxetine (PRX, C_19_H_20_FNO_3_), as a selective serotonin reuptake inhibitor, is the first‐line drug for the treatment of depression. However, it has obvious toxic and side effects on male sexual function (Ulker et al., 2020). Many reports have shown that continuous administration of paroxetine leads to male sexual dysfunction by causing damage to testicular tissue, decreased sperm quality, decreased libido, and ED in patients (Ademosun et al., 2022; Erdemir et al., 2014; Xu et al., 2022). Therefore, PRX is often used as a modeling drug to construct animal models of male sexual dysfunction to explore the pathogenesis of male sexual dysfunction and the ameliorative effects of related natural active substances on this disease (Canpolat et al., 2022; Saikia et al., 2023).

Bioactive peptides from marine organisms have been shown to have various biological activities, such as improving immunity, anti‐skin photoaging, anti‐oxidation, and improving reproductive disorders (Cheung et al., 2015; Li, Yang, et al., 2022; Li, Zhang, et al., 2022; Zhang et al., 2019; Zhang, Lv, et al., 2022; Zhang, Zhang, et al., 2022). Among them, oyster peptide (OP) is one of the most biologically active peptides to improve male reproductive disorders. In previous studies, Zhang, Peng, et al. (2021); Zhang, Wei, et al. (2021) found that OP can promote the synthesis and secretion of androgen and nitric oxide (NO) in mouse Leydig cells. Luo et al. (2021) also found that OP can improve the sexual ability of male mice after fatigue swimming. Our previous studies have also confirmed that the hydrolysate of oyster peptides from Hong Kong has a potential protective effect on triptolide‐induced reproductive disorders in male mice (Zhang, Peng, et al., 2021; Zhang, Wei, et al., 2021). This shows that OP has a great potential to improve male sexual dysfunction. However, there are still relatively few studies on the use of OP as the main raw material in actual food research and development.

*Pfaffia glomerata*, Amaranthaceae, of the *Faffia* genus, native to South America, has been successfully introduced and cultivated in Jiangxi Province, Guangdong Province, and other places in China. It is rich in triterpenoid saponins, steroids, trace elements, and other active ingredients. It has biological activities, such as aphrodisiac, anti‐fatigue, anti‐tumor, and so on (Lu et al., 2018). In 1994, the US Food and Drug Administration (FDA) approved *Pfaffia glomerata* as a healthy food. At the same time, *Pfaffia glomerata* is also used as a nutritional supplement for athletes because of its rich biologically active ingredients to improve athletes' tolerance and resistance to external pressure (Vardanega et al., 2017; Zhao et al., 2022). In addition, *Pfaffia glomerata* extract (PGE) also has the effect of enhancing the sexual ability of impotent rats, promoting the production of sex hormones in male mice, and stimulating the penis tissue of Swiss mice (Arletti et al., 1999; Dias et al., 2020; Oshima & Gu, 2003). Therefore, PGE also has great potential in improving male reproductive dysfunction. However, there are still relatively few products developed to improve male sexual dysfunction with PGE as the main raw material.

As a popular product, pressed candy (PC) is easy to carry in daily consumption and is widely accepted by the market and consumers. Therefore, this study aims to use OP and PGE as the main raw materials to develop a pressed candy with high nutritional value and improved male sexual dysfunction, so as to meet people's increasing demand for nutritious and healthy food. At the same time, it promotes the development of OP and PGE deep processing industry and provides a new way for the development of OP and PGE products.

## 2 MATERIALS AND METHODS

### 2.1 Experimental materials and excipients

Oyster peptide was purchased from the Chengmai Branch of Hainan Shengmeinuo Biotechnology Co., Ltd. (Haikou, China). *Pfaffia glomerata* (more than 3 years and less than 5 years) was purchased from Guangdong Leizhou Peninsula Forest Economic and Technological Innovation Center (Zhanjiang, Guangdong), identified by Professor Yu Liying (Guangxi Zhuang Autonomous Region Medicinal Botanical Garden and The Institute of Medicinal Plant Development). Microcrystalline cellulose and magnesium stearate were purchased from Huzhou Linghu Xinwang Chemical Co., Ltd. (Huzhou, China). Mannitol was purchased from Shijiazhuang Ruixue Pharmaceutical Co., Ltd. (Shijiazhuang, China). Lactose was purchased from Zhengzhou Jieshang Biotechnology Co., Ltd. (Zhengzhou, China). Skim milk powder was purchased from Maxigenes Pty Ltd. (New South Wales, Australia). Siraitia grosvenorii glycosides were purchased from Hunan Huacheng Biological Resources Co., Ltd. (Changsha, China). Citric acid was purchased from Weifang Yingxuan Industrial Co., Ltd. (Weifang, China). Peppermint essence and vanillin were purchased from Shenzhen Chenxin Flavor & Fragrance Co., Ltd. (Shenzhen, China). Oleanolic acid was purchased from Shanghai McLean Biotechnology Co., Ltd. (Shanghai, China).

### 2.2 Experimental drugs

Paroxetine hydrochloride tablets are provided by Sino‐US Tianjin Shike Pharmaceutical Co., Ltd. (Tianjin, China). Sildenafil citrate tablets are provided by Pfizer Pharmaceutical Co., Ltd. (Dalian, China). Other chemicals or reagents are of analytical grade.

### 2.3 Preparation of PGE and determination of PC saponin content

The preparation method of PGE and the determination method of PC saponin content were carried out according to the previous research methods of our research group (Huang et al., 2023). In simple terms, 300 g of *Pfaffia glomerata* powder was ultrasonically extracted with 9000 mL of 70% ethanol (1:30 w/v, 350 W, 60°C, 1 h) (KQ‐500DB, Kunshan Ultrasonic Instruments Co., Ltd., Kunshan, China), cooled, and centrifuged (4°C, 13,881 *g* (revolutions per minute), 15 min) (Lynx 6000, Thermo Fisher Scientific, Waltham, MA, USA) to obtain the supernatant. Then the filter residue was extracted again under the same conditions. After the two supernatants were combined, PGE was obtained by concentration and freeze‐drying procedures. The content of saponins in PC was determined by vanillin–perchloric acid–glacial acetic acid method. Two milligrams of oleanolic acid standard and 0.3 g of pretreated PC were accurately weighed, respectively, and 0.2 mg/mL standard solution and a certain concentration of sample solution were prepared by methanol solution. The absorbance values of the standard and sample were determined at 545 nm using a microplate reader (Varioskan Flash, Thermo Fisher Scientific, Waltham, USA), and the saponin content was finally calculated. The experiment was repeated three times.

### 2.4 Oyster peptide and *Pfaffia glomerata* pressed candy processing process

Raw materials and excipients were mixed in proportion → over 80‐mesh sieve granulation (standard screen, GB/T6003.1‐2022, Shaoxing Shangyu Shengchao Instrument Equipment Co., Ltd.) → reparation of soft materials → over 14‐mesh sieve granulation (standard screen, GB/T6003.1‐2012, Shaoxing Shangyu Shengchao Instrument Equipment Co., Ltd.) → drying at 60°C for 3 h (GZX‐9246, Shanghai, China) → over 14‐mesh sieve granulation → pressing tablets (TDP‐0T, Guangzhou, China) → quality inspection → packaging → finished product.

### 2.5 One‐way experiment design

According to the previous animal experimental results of our research group, the ratio of OP to PGE was fixed at 2:3 (Huang et al., 2023; Zhang, Lv, et al., 2022; Zhang, Zhang, et al., 2022). On the basis of the previous pre‐experiment, the addition amount of the main raw material was fixed at 25%, the skimmed milk powder was 2%, the vanillin was 2%, the siraitia grosvenorii glycosides was 0.2%, the citric acid was 0.2%, the mint essence was 2%, and the magnesium stearate was 1.5%. With the comprehensive score as the index, the addition amount of filler (microcrystalline cellulose addition amounts were 10%, 15%, 20%, 25%, and 30%; D‐mannitol addition amounts were 10%, 15%, 2%, 25%, and 30%; and lactose addition amounts were 20%, 25%, 30%, 35%, and 40%) and the effects on the quality of pressed candy were studied.

### 2.6 D‐optimal mixture experiment design

On the basis of one‐way experiment, according to the experimental design software Design Expert 8.0.6 D‐optimal mixture's experimental design principle, microcrystalline cellulose (A), D‐mannitol (B), and lactose (C) were selected as experimental factors. The hardness (α1), friability (α2), and tablet weight variation (α3) were used as the determination indexes. The comprehensive score was taken as the response value to investigate the effect of filler addition on the comprehensive score of PC. The factor level coding table is shown in Table 1.

**TABLE 1:** Design factors and levels of D‐optimal mixture experiment.

Coding	Experimental factors	Levels	
		0	1
A	Microcrystalline cellulose/%	10	20
B	Mannitol/%	15	25
C	Lactose/%	25	35

### 2.7 Determination method of PC tablets' characteristics

Referring to the method of Lai et al. (2017), 20 samples were taken and put into the tablet multipurpose tester (Shanghai Huanghai Pharmaceutical Testing Instrument Co., Ltd., Shanghai, China) to determine the hardness, and finally the average value was taken. The friability, tablet weight variation, and disintegration time of pressed candy were determined according to the relevant rules of ‘Pharmacopoeia of the People's Republic of China (National Pharmacopoeia Committee, 2020)’.

### 2.8 Comprehensive score calculation

Referring to the method of Lai et al. (2017), the process parameters of pressed candy were optimized by multi‐index comprehensive scoring method with hardness (α1), friability (α2), and tablet weight variation (α3) as the determination indexes. The comprehensive score is the sum of the scores of each index, that is, comprehensive score (Y) = α1 + α2 + α3, and the scoring criteria are shown in Table 2.

**TABLE 2:** Multi‐index comprehensive score table.

Coding	Index name	Index unit	Scoring method
α_1_	Hardness	*N*	0.1 times the value
α_2_	Friability	%	10 times the reciprocal of the value
α_3_	Tablet weight variation	%	10 times the reciprocal of the value

### 2.9 Determination of basic components

The crude protein content of the samples was determined by Kjeldahl method according to the national standard of the People's Republic of China GB 5009.5‐2016. The crude fat content was determined by Soxhlet extraction method according to the national standard of the People's Republic of China GB 5009.6‐2016. The moisture content of the sample was determined by atmospheric drying method according to the national standard of the People's Republic of China GB 5009.3‐2016. The ash content was determined by high temperature burning method according to the national standard of the People's Republic of China GB 5009.4‐2016. The total sugar content was determined by phenol–sulfuric acid method according to the national standard of the People's Republic of China GB/T 9695.31‐2008.

### 2.10 Determination of free amino acids and trace elements

The free amino acid content of PC was determined according to the national standard of the People's Republic of China GB/T 22729‐2008 “Marine fish oligopeptide powder”. The contents of trace elements zinc (Zn) and selenium (Se) in PC were determined by inductively coupled plasma mass spectrometry (ICP‐MS) according to the national standard of the People's Republic of China GB/T5009.268‐2016.

### 2.11 Animal experimental design

Sixty‐four 5‐week‐old specific pathogen‐free (SPF) ICR mice [(26 ± 2) g], half male and half female, were provided by Guangzhou Yancheng Biotechnology Co., Ltd. (Beijing, China) with an animal license number of (SCXK (Beijing) 2019‐0010). All mice were maintained at 24 ± 2°C, relative humidity (RH) of 50% – 60%, and illumination for 12 h every day. Before the formal experimental procedure, all mice were free to eat and drink water for 7 days. The 32 mice were randomly divided into 4 groups (*n* = 8): Control group (CN, equal volume of 1% sodium carboxymethyl cellulose and distilled water), PRX group (treated 14 mg/kg paroxetine), PRX + SDF group (treated 14 mg/kg paroxetine +7 mg/kg sildenafil), and PRX + PC group (treated 14 mg/kg paroxetine +150 mg/kg pressed candy (calculated as PGE, equivalent to 1000 mg/kg pressed candy)). The intragastric doses of PRX and SDF referred to the study of Huang et al. (2023). PRX, SDF, and PC were suspended in 1% sodium carboxymethyl cellulose, and PRX calculated as C_19_H_20_FNO_3_, SDF calculated as C_22_H_30_N_6_O_4_S·C_6_H_8_O_7_. All mice were gavaged orally for 28 days. The whole animal experiment was approved by the Animal Experimental Ethics Committee of Guangdong Ocean University (China (GDOU‐LAE‐2022‐039)).

### 2.12 Sexual behavior experiment

One hour after the last oral gavage, the mice were made to perform sexual behavior experiments. At 48 h and 4 h before the formal experiment, female mice were intramuscularly injected with 0.2 mg/mouse estradiol benzoate injection and 1 mg/kg progesterone injection to make female mice enter estrus (Huang et al., 2023; Khalid et al., 2022). The experiment was arranged in a quiet, dark red‐light room at 20: 00–24: 00 p.m. Male mice were first placed in transparent cages of 30 cm × 15 cm × 15 cm for 15 min, followed by camera recording. Sexual behavioral parameters were observed and recorded for the first 30 min, including mount latency (the time of the first mounting behavior in male mice, ML), intromission latency (the time of first intromission of male mice into the female vagina, intromission latency [IL]), mount frequency (the number of male mice mounting behavior within 30 min, MF), and intromission frequency (total number of intromissions in male mice within 30 min, intromission frequency [IF]) (Canpolat et al., 2022; Toyin & Olaide, 2012).

### 2.13 Determination of serum hormones and biochemical indicators

At the end of the experiment, according to the relevant provisions of the Ethics Committee of Guangdong Ocean University, male mice were anesthetized by intraperitoneal injection of 1% pentobarbital sodium (50 mg/kg), the eyeballs were removed for blood collection, and then the spine was dislocated and sacrificed. After blood coagulation, serum was collected by centrifugation at 4°C, 1952 *g* for 15 min (UNIVER 320 R, Tuttlingen, Germany) according to Chen, Li, et al. (2019); Chen, Shi, et al. (2019) method. Serum testosterone (T) and luteinizing hormone (LH) levels were measured by enzyme‐linked immunosorbent assay (ELISA) kit (Jiangsu Meimian Industrial Co., Ltd, Yancheng, China). Partial penile corpus cavernosum tissue homogenate and right testis tissue homogenate were prepared according to the kit instructions, respectively, and the supernatant was collected. Their protein content was determined using a bicinchoninic acid assay (BCA) protein kit (Beyotime Biotechnology, Shanghai, China). At the same time, the content of nitric oxide (NO) (Nanjing Jiancheng, Nanjing, China) and cyclic guanosine monophosphate (cGMP) (Jiangsu Meimian Industrial Co., Ltd., Yancheng, China) in penis was determined. Superoxide dismutase (SOD), catalase (CAT), and glutathione peroxidase (GSH‐Px) activities and malondialdehyde (MDA) content in testes were also measured by kits (Nanjing Jiancheng, Nanjing, China).

### 2.14 Histopathology analysis

The left testicular tissue and the posterior 1/3 of the penis tissue of the mice were fixed with paraformaldehyde fixative for more than 24 h, respectively, and then subjected to gradient alcohol dehydration and paraffin immersion. Then the paraffin‐immersed tissue was embedded and sliced at a thickness of 4 μm. Subsequently, the sections were deparaffinized and stained with hematoxylin and eosin (H&E) (G1003, Servicebio, Wuhan, China) (Shon et al., 2024). Finally, images of testicular tissue and penile tissue were collected and analyzed using an upright optical microscope (Nikon Eclipse E100, Tokyo, Japan).

### 2.15 Determination of microbial and heavy metal content indicators

The determination of microbial indexes in pressed candy was carried out according to GB 17399‐2016 ‘national food safety standard candy’ and GB 29921‐2013 ‘national food safety standard food pathogenic bacteria limit’ standards. The determination of heavy metal content was carried out according to GB 2762–2017 ‘National Food Safety Standard Food Contaminant Limits’.

### 2.16 Statistical analysis

All the results of the experiments are expressed as the mean ± standard deviation (SD) and analyzed and plotted using SPSS 27 and GraphPad 9.3 software. All data were analyzed using one‐way analysis of variance (ANOVA), and with a statistically significant *p* < .05.

## 3 RESULTS

### 3.1 Analysis of one‐way experiment

**FIGURE 1:**
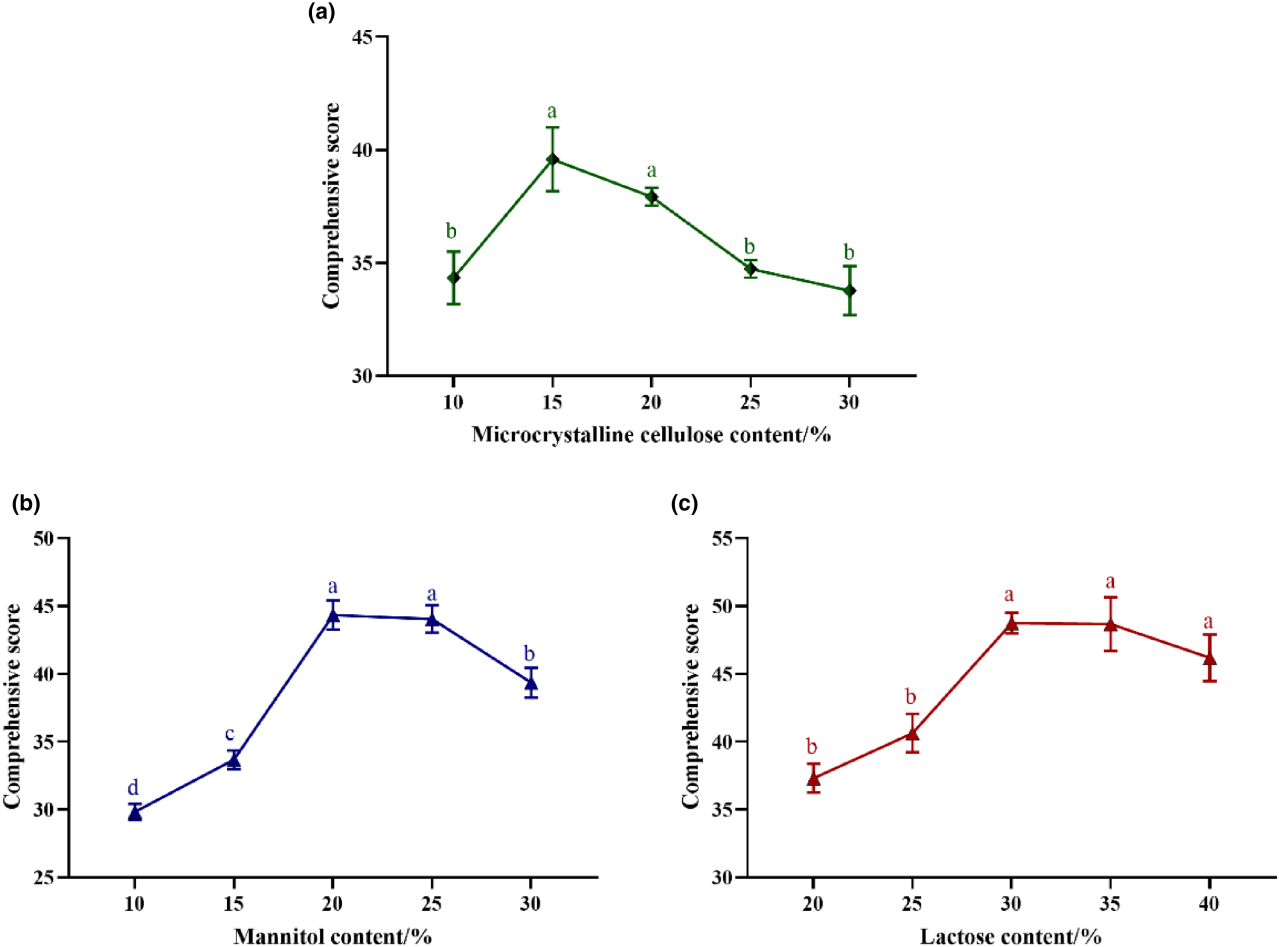
Effect of filler content on PC comprehensive score. Lowercase letters with different superscripts show significant differences (*p* < .05).

From Figure 1a–c, we observe that with the increase in the amount of microcrystalline cellulose added, the comprehensive score of the PC showed a trend of increasing first and then decreasing, and the comprehensive score was the highest when the addition amount was 15%. When the amount of mannitol added increased to 20%, the comprehensive score of the PC was the highest. In addition, with the increase of lactose addition, the comprehensive score of PC also increased first and then tended to decline gently. When the lactose addition was 30%, the comprehensive score of PC reached the highest value. Therefore, according to the results of single factor test, the amount of microcrystalline cellulose added was 15%, the amount of mannitol added was 20%, and the amount of lactose added was 30% as the intermediate level of D‐optimal mixture experiment design factors.

### 3.2 D‐optimal mixture experiment analysis

According to the results of one‐way experiment, the comprehensive score was used as the response value, and the formula of PC was optimized according to the principle of D‐optimal mixture design. The experimental design scheme and result analysis are shown in Table 3, and the analysis of variance results are shown in Table 4. The regression variance model of the comprehensive score of PC is (Y) = 28.02*A + 33.29*B + 31.91*C + 72.05*A*B + 64.14*A*C + 1.23*B*C + 245.16*A*B*C. The model has a significant difference (*p* < .05), the lack of fit was not significant (*p* > .05), the correlation coefficient *R* ^2^ = .9631, and the correction correlation coefficient $R_{\mathrm{Adj}}^{2}$ = .9385, indicating that the regression equation model fits well with the actual experimental data and can predict the related properties of the pressed candy.

**TABLE 3:** Design scheme and results of D‐optimal mixture experiment.

Number	Microcrystalline cellulose content (A)/%	Mannitol content (B)/%	Lactose content (C)/%	Hardness/*N*	Fragility/%	Tablet weight variation/%	Comprehensive score (Y)
1	20.0	20.0	30.0	70.72	0.46	2.8	32.38
2	20.0	21.7	28.3	72.44	0.46	2.4	33.15
3	20.0	20.0	30.0	75.22	0.45	2.7	33.45
4	20.0	15.0	35.0	78.32	0.49	2.4	32.41
5	20.0	15.0	35.0	80.48	0.45	2.2	34.82
6	20.0	25.0	25.0	67.83	0.48	2.5	31.62
7	18.3	18.4	33.3	77.09	0.31	2.1	44.73
8	16.7	21.7	31.7	81.25	0.24	1.5	56.46
9	16.7	25.0	28.3	76.53	0.37	1.1	43.77
10	15.0	20.0	35.0	81.54	0.28	1.4	51.01
11	15.0	25.0	30.0	78.52	0.33	1.3	45.85
12	15.0	20.0	35.0	83.08	0.31	1.8	46.12
13	15.0	25.0	30.0	79.19	0.32	1	49.17
14	15.0	20.0	35.0	82.04	0.29	1.5	49.35
15	13.3	25.0	31.7	75.07	0.42	1.2	40.84
16	10.0	25.0	35.0	68.45	0.57	2.5	28.39

**TABLE 4:** Analysis of variance of pressed candy D‐Optimal mixture experiment design.

Source of variance	Quadratic sum	Degree of freedom	Mean square	*F* value	Pr > *F*	Significance
Model	1058.2	6	176.37	39.15	<0.0001	**
Linear regression	127.6	2	63.8	14.16	0.0017	**
AB	467.91	1	467.91	103.9	<0.0001	**
AC	311.65	1	311.65	69.18	<0.0001	**
BC	0.12	1	0.12	0.027	0.8722	
ABC	76.13	1	76.13	16.9	0.0026	**
Residual error	40.54	9	4.5			
Lack of fit	19.19	4	4.8	1.12	0.4395	
Pure error	21.35	5	4.27			
Total regression	1098.75	15				
*R* ^2^ = .9631			$R_{\mathrm{Adj}}^{2}$ = .9385			

** *p* < .01.

According to the results of pre‐experiment and D‐Optimal mixture experiment design, the optimal process formula of PC was as follows: 25% raw material, 16% microcrystalline cellulose, 21.8% mannitol, 32.3% lactose, 2% vanillin, 0.2% siraitia grosvenorii glycosides, 0.2% citric acid, 2% peppermint flavoring, 2% skimmed milk powder, and 1.5% magnesium stearate. The PC prepared under this process formula has suitable taste, complete tablet, and smooth surface.

### 3.3 Analysis of the basic components and saponin content of PC

According to the above‐mentioned optimal process formula, PC was prepared. From Table 5, we notice that the crude protein content of PC was 8.10%, while the crude fat content was only 0.55%, indicating that PC had the characteristics of high protein and low fat. In addition, the saponin content of PC is 1.88%. Some scholars have found that triterpenoid saponins are one of the main active components of *Pfaffia glomerata*, which have the effects of anti‐fatigue and promoting androgen synthesis and secretion (Liu et al., 2022; Oshima & Gu, 2003). Our research group used ultra‐high liquid chromatography–mass spectrometry (UPLC–MS) to characterize the structure of PGE in the early stage, and found that it mainly contained nine triterpenoids (20‐Hydroxyecdysone, Achyranthoside E, Chikusetsusaponin IVA, and so on.), and verified the improvement effect of PGE on reproductive dysfunction in male mice (Huang et al., 2023). Therefore, it can be preliminarily judged that PC has the potential to improve male sexual disorders.

**TABLE 5:** Analysis of basic components and saponin content of pressed candy (%).

Item	Moisture	Crude protein	Crude fat	Total sugar	Ash	Saponin
Pressed candy	2.10 ± 0.02	8.10 ± 0.30	0.55 ± 0.07	56.29 ± 1.04	2.21 ± 0.06	1.88 ± 0.039

### 3.4 Analysis of PC free amino acid content and trace elements

The composition and content of free amino acids in PC are shown in Table 6. PC amino acids are complete and contain essential amino acids (EAA) required for human growth and development. Among them, essential amino acids (EAA) account for 48.91% of total amino acids (TAA), which is much higher than the minimum dietary intake recommended by the World Health Organization (WHO) (Wu, 2016). Studies have shown that appropriate supplementation of EAA can promote fertility enhancement. On the contrary, long‐term insufficient intake of EAA will lead to spermatogenesis disorders and decreased fertility (Hou & Wu, 2018). In addition, hydrophobic amino acid (HAA) in PC accounts for 36.81% of TAA. To a certain extent, HAA supplementation can alleviate oxidative stress injury in the body (Sarmadi & Ismail, 2010). PC also contains a high proportion of branched‐chain amino acids (BCAA). Studies have shown that BCAA can provide energy for the body (de Bisschop et al., 2021). It also plays a powerful role in maintaining spermatogenesis and sex hormone synthesis and secretion (Bahadorani et al., 2019). The results showed that PC had high nutritional value.

**TABLE 6:** Analysis of PC free amino acid content (g/100 g).

Number	Free amino acid	Content
1	Asp	0.056 ± 0.016
2	Glu	0.105 ± 0.02
3	Ser	0.031 ± 0.008
4	His	0.042 ± 0.013
5	Gly	0.036 ± 0.004
6	Thr^a^	0.078 ± 0.004
7	Arg	0.279 ± 0.021
8	Ala^b^	0.105 ± 0.043
9	Tyr	0.102 ± 0.013
10	Cys‐s	0.009 ± 0.009
11	Val^abc^	0.059 ± 0.012
12	Met^ab^	0.027 ± 0.005
13	Phe^ab^	0.096 ± 0.004
14	Ile^abc^	0.084 ± 0.006
15	Leu^abc^	0.176 ± 0.007
16	Lys^a^	0.220 ± 0.017
17	Pro^b^	0.009 ± 0.006
	Total amino acid, TAA	1.51
	Essential amino acid, EAA	0.74
	Hydrophobic amino acid, HAA	0.56
	Branched‐chain amino acid, BCAA	0.32
	EAA/TAA (%)	48.91
	HAA/TAA (%)	36.81
	BCAA/TAA (%)	21.08

*Note*: a is essential amino acid; b is hydrophobic amino acid; and c is branched‐chain amino acid.

The content of zinc (Zn) in PC was 0.607 mg/100 g. It has been reported that in addition to maintaining normal physiological and biochemical functions and improving sperm quality in the human body, Zn also plays an important role in the development of the testis and the synthesis and secretion of androgens (Santos & Teixeira, 2019). Therefore, appropriate supplementation of Zn has a certain protective effect on the male reproductive system. At the same time, the content of selenium (Se) in PC is 0.149 mg/kg. In addition to its strong antioxidant effect, Se is also indispensable in the biosynthesis of T in human and animal reproduction and the production of sperm (Adedara et al., 2019). It can be seen that Se is also essential for male sexual health.

### 3.5 Effect of PC on sexual behavior in male mice

**FIGURE 2:**
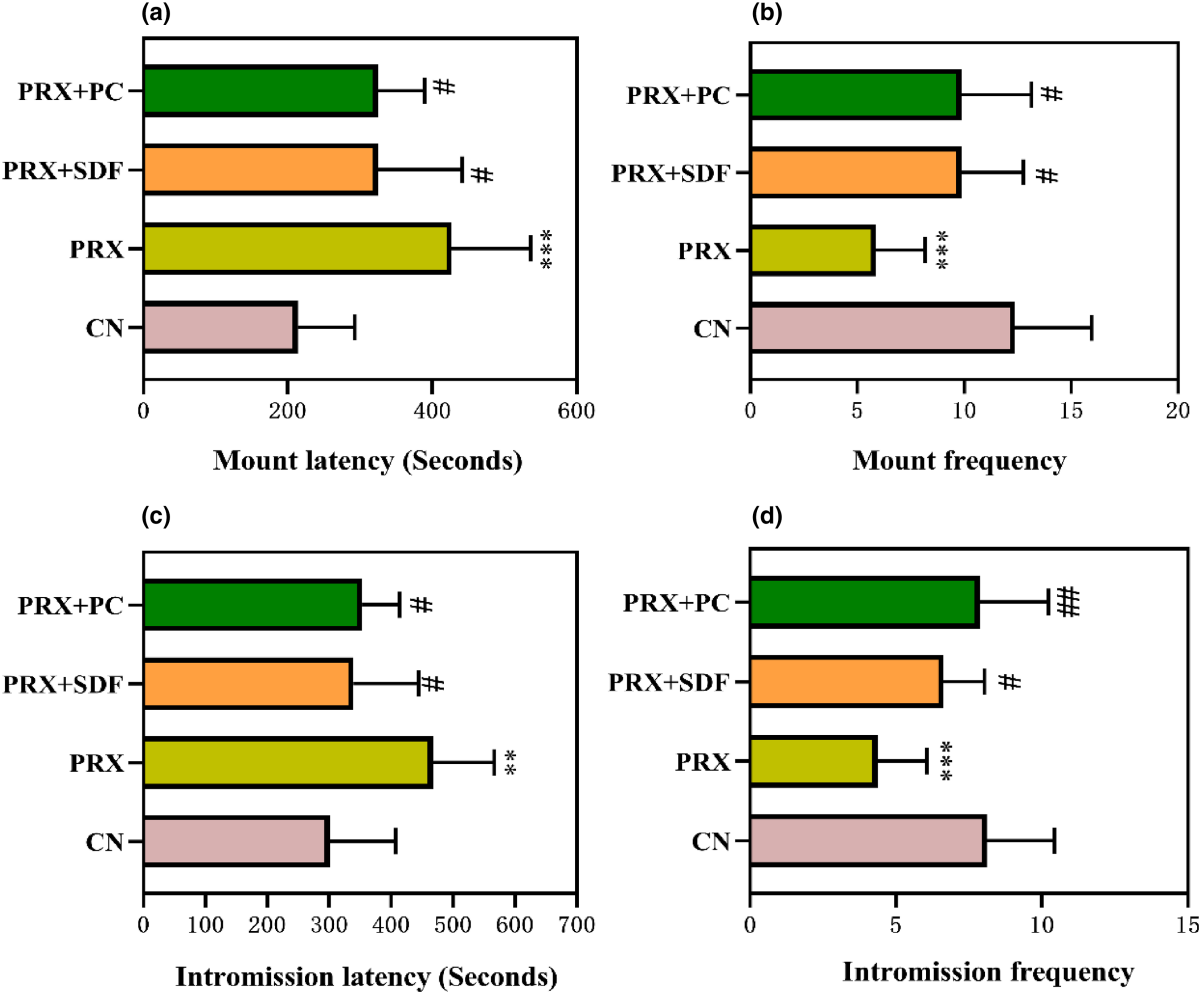
Effects of PC on sexual behavior in male mice (a. ML; b. MF; c. IL; and d. IF). The data were expressed as mean ± SD, *n* = 8. Compared with the CN group, *means *p* < .05, ** means *p* < .01, and ***means *p* < .001. Compared with the PRX group, # means *p* < .05; ## means *p* < .01, and ### means *p* < .001.

Sexual behavior index is an important index to reflect the sexual ability of male mice. As shown in Figure 2, the ML and IL of the PRX group were 2 times and 1.6 times those of the CN group, respectively, which significantly prolonged ML (*p* < .001) and IL (*p* < .001), while MF and IF decreased by 53% and 46% compared with the CN group, indicating that PRX administration resulted in a decrease in the sexual ability of male mice. After PC intervention, ML and IL (*p* < .01) of male mice were significantly shortened by 23% (*p* < .05) and 24% compared with the PRX group. In addition, compared with the PRX group, the PRX + PC group also significantly increased the MF and IF of male mice by 68% (*p* < .01) and 80% (*p* < .05), respectively. The results showed that PC intervention could effectively improve PRX‐induced sexual ability decline in male mice.

### 3.6 Effects of PC on testicular tissue and serum sexual hormones

**FIGURE 3:**
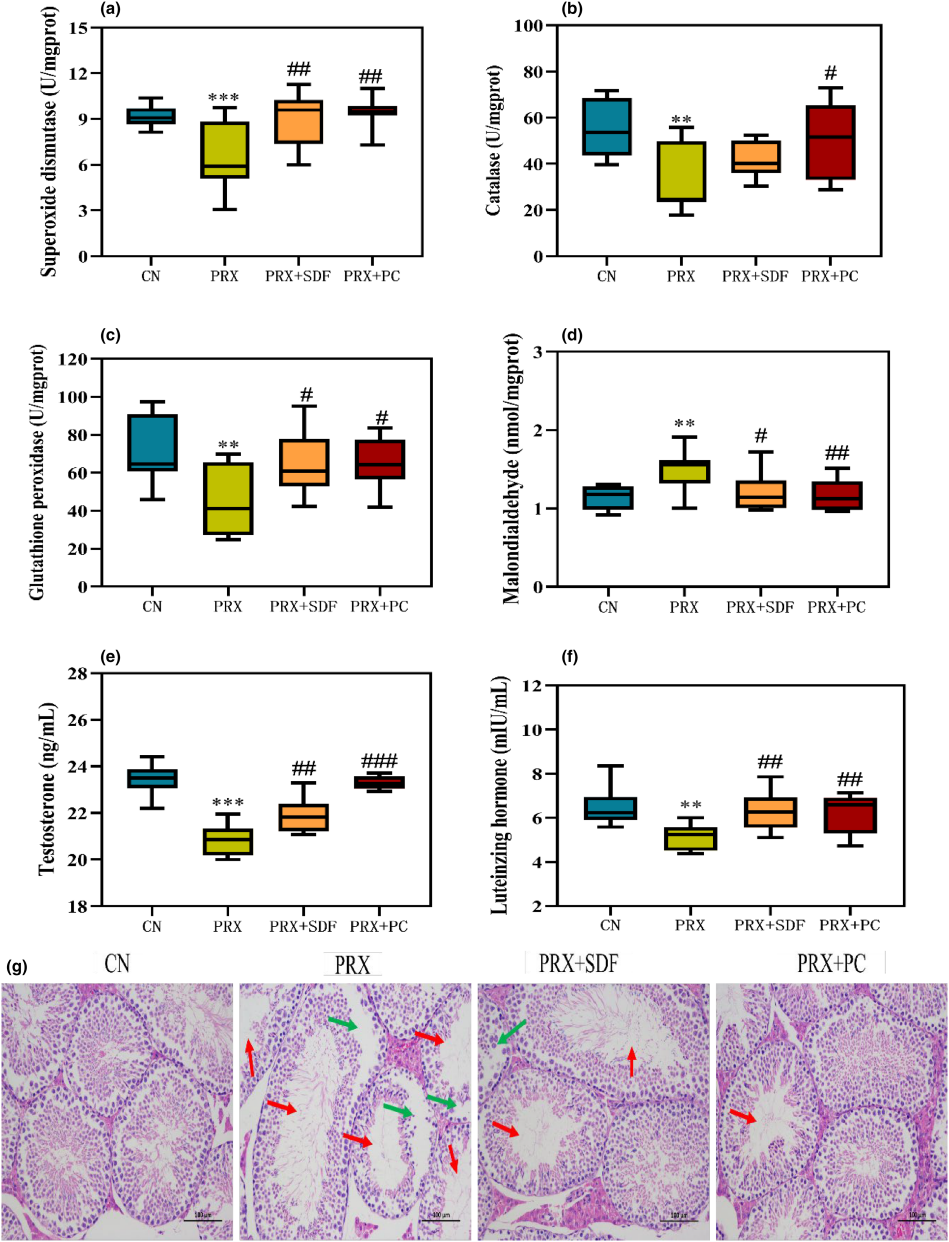
Effects of PC on testicular tissue and serum sexual hormones (a. SOD; b. CAT; c. GSH‐Px; d. MDA; e. T; and f. LH). The data were expressed as mean ± SD, *n* = 8. Compared with the CN group, *means *p* < .05, **means *p* < .01, and ***means *p* < .001. Compared with the PRX group, #means *p* < .05; ## means *p* < .01, and ### means *p* < .001. (g) Histopathological analysis. The red arrow means the number of sperm is reduced, and the green arrow means the vacuolization is serious.

Oxidative stress occurs when the pro‐oxidant in the body is stronger than the antioxidant, which may cause damage to lipids, nucleic acids, and proteins in the body's cells, thereby damaging the reproductive system. According to Figure 3a–c, it shows that the activities of SOD, CAT, and GSH‐Px in testicular tissue of the PRX group were significantly lower than those of the CN group by 34% (*p* < .001), 39% (*p* < .01), and 37% (*p* < .01), respectively. However, compared with the PRX group, the SOD, CAT, and GSH‐Px activities of testicular tissue were significantly increased by 43% (*p* < .01), 51% (*p* < .05), and 44% (*p* < .05) after PC intervention, respectively, indicating that PC has good antioxidant capacity. In addition, MDA is a product of lipid peroxidation, and its accumulation can damage the structure of testicular tissue and damage germ cells, leading to male reproductive disorders (Famurewa et al., 2023). It can be seen from Figure 3d that the content of MDA in testis tissue of the PRX group was 31% higher than that of the CN group (*p* < .01). Further analysis of testicular histopathological results (Figure 3g) showed that the testicular tissue structure of CN group mice was good, while the number of germ cells and sperm in the seminiferous tubules of mice in the PRX group decreased sharply, the cells were irregularly arranged, and the seminiferous tubules were severely vacuolated. Therefore, it is inferred that PRX may lead to oxidative stress injury of testicular tissue, thus destroying the structure of testicular tissue. Testicular tissue is the main place for male sexual hormone synthesis and secretion (Li, Yang, et al., 2022; Li, Zhang, et al., 2022), and sexual hormone levels largely determine the normal growth and development ability and sexual ability of male. From Figure 3e,f, it can be seen that PRX administration significantly reduced the serum T and LH content of male mice (*p* < .001), which may be related to the damage of testicular tissue, the main organ producing male sexual hormone. This may also be consistent with the decline in sexual behavior of male mice mentioned in the previous section. However, compared with the PRX group, the testicular tissue morphology of male mice was improved after PC intervention, and the T content and LH content were increased by 12% (*p* < .01) and 22% (*p* < .01), respectively. Obviously, PC intervention alleviated the oxidative stress injury of testicular tissue caused by PRX, thus improving the synthesis and secretion of male sex hormones.

### 3.7 Effects of PC on histopathological results and NO/cGMP signaling pathway in penile tissue of male mice

**FIGURE 4:**
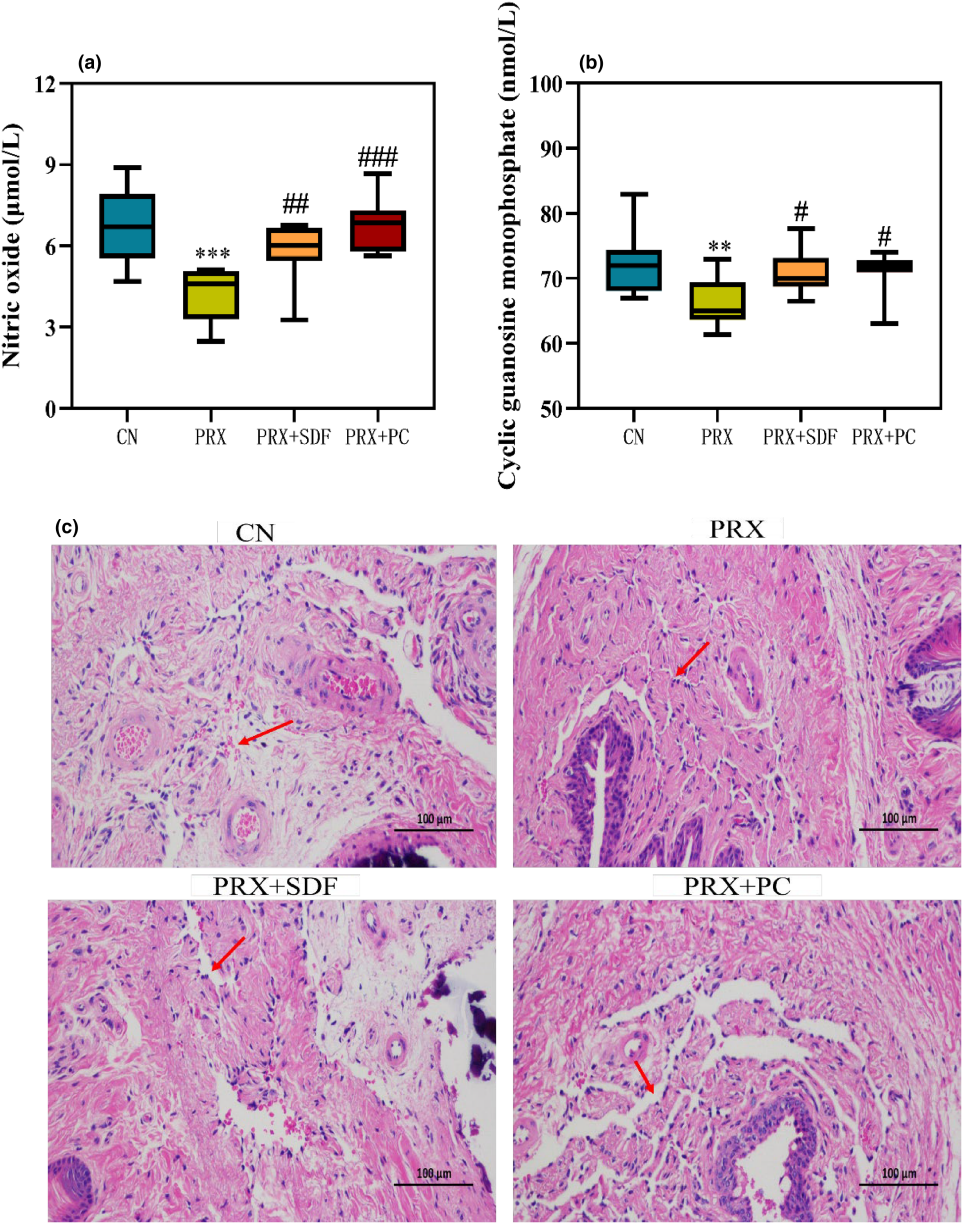
Effect of PC on penis tissue of male mice (a. NO; b. cGMP). The data were expressed as mean ± SD, *n* = 8. Compared with the CN group, *means *p* < .05, **means *p* < .01, and ***means *p* < .001. Compared with the PRX group, #means *p* < .05; ## means *p* < .01, and ### means *p* < .001. (c) Penile histopathological analysis. The red arrow indicates the cavernous sinus.

In addition to sex hormone levels, the NO/cGMP signaling pathway also plays a key role in male reproductive function, which is mainly involved in male sexual function by mediating penile erection (Akintunde et al., 2023). As shown in Figure 4a,b, PRX administration significantly decreased the NO content and cGMP content in the penile tissue of male mice by 38% (*p* < .001) and 9% (*p* < .01), respectively, compared with the CN group. However, compared with the PRX group, the content of NO and cGMP in PRX + PC group increased by 61% (*p* < .001) and 7% (*p* < .05), respectively. NO/cGMP‐mediated can reduce the concentration of Ca^2+^ in smooth muscle cells, thereby promoting penile congestion and erection (Yadav & Mishra, 2024). Further analysis of the histopathological results of the penis (Figure 4c) showed that the penile tissue of the CN group was normal, the cavernous sinus was abundant, and some red blood cells were visible in the sinus cavity. In the PRX group, there were pathological changes such as blood sinus reduction and stenosis. However, SDF and PC intervention reduced the pathological damage of the corpus cavernosum in mice, and the blood sinuses increased and widened. The results showed that PC intervention has a good improvement effect on ED caused by PRX.

### 3.8 PC tablet characteristics, microbial and heavy metal content analysis

The tablet characteristics can directly reflect the quality of PC. The PC was prepared under the optimal process formula and its tablet characteristics were determined. The results are shown in Table 7. The tablet weight variation, hardness, friability, and disintegration time of PC all meet the standards required by the Pharmacopoeia of the People's Republic of China.

**TABLE 7:** Characteristics of PC.

Items	Tablet weight variation/%	Hardness/N	Fragility/%	Disintegration time/min
Pressed candy	1.000 ± 0.018	79.92 ± 1.92	0.31 ± 0.0176	8.39 ± 0.5956
Whether composite standard	Qualified	Qualified	Qualified	Qualified

The contents of microorganisms and heavy metals in PC were determined according to the relevant methods specified in the national food safety standards. The results are shown in Table 8. The measured microbial indicators and heavy metal contents were in line with the national food safety standards.

**TABLE 8:** Analysis of microorganisms and heavy metals in PC.

Items	Unit	Test results
Total number of bacterial colonies	CFU/g	<10
Coliform group	CFU/g	<10
Salmonella	/25 g	Not detected
Shigella spp.	/25 g	Not detected
Staphylococcus aureus	/25 g	Not detected
Streptococcus hemolyticus	/25 g	Not detected
Fungus	CFU/g	<10
Yeast	CFU/g	<10
Pb	mg/kg	Not detected
As	mg/kg	0.18
Cd	mg/kg	0.042
Hg	mg/kg	Not detected
Cr	mg/kg	0.31
Total hexachlorocyclohexane	mg/kg	Not detected
Total DDT	mg/kg	Not detected

## 4 DISCUSSION AND CONCLUSIONS

The amount of filler added is an important factor affecting the indicators of tablets. Therefore, the amount of filler added needs to be controlled when preparing tablets. When the proportion of tablet fillers (microcrystalline cellulose, mannitol, lactose) is different, the values of each index of the tablet are quite different. When the dosage is small, the hardness is not enough, the tablet is easy to break, the weight of the tablet is quite different, and the disintegration time of the tablet is not ideal when the dosage is large (Lai et al., 2017). On the basis of one‐way experiment, combined with D‐optimal mixture experiment, the optimum addition amount of PC filler was optimized with comprehensive score as index. The PC prepared under this process formula has suitable taste, complete tablet, smooth surface, and high nutritional value. The hardness, friability, tablet weight variation, disintegration time limit, and microbial and heavy metal content of PC were in line with the standards of Chinese Pharmacopoeia and Chinese national food safety standards.

Arginine (Arg) is an indispensable EAA in the human body. By measuring the content of free amino acids in PC, it was found that the content of Arg was the highest. Studies have reported that Arg can enhance the sperm motility and quantity of oligoasthenospermia men, and promote the synthesis and secretion of T in Leydig cells (Yang et al., 2019). NO is an important product synthesized by NOS in the process of L‐Arg catabolism, which plays a key role in male reproductive function, including spermatogenesis, sperm motility, sperm capacitation, penile erection, and so on (El‐Shalofy et al., 2023). NO mainly maintains penile erection by regulating cGMP concentration in the corpus cavernosum (2023; Chen, Li, et al., 2019; Chen, Shi, et al., 2019). In this study, the concentration of NO in the PRX group was significantly lower than that in the CN group, which may be due to the increase of arginase activity in mice caused by PRX, thus inhibiting the concentration and bioavailability of NO. This finding was verified in the study of Ademosun et al. (2019). However, after PC intervention, the concentration of NO was significantly increased, which may be due to the role of PC as a precursor of NO synthesis or an arginine enzyme inhibitor. Phosphodiesterase‐5 (PDE‐5) is an enzyme that hydrolyzes cGMP (Huang et al., 2020). In previous studies, we also found that triterpenoids in *Pfaffia glomerata* can act as PDE‐5 inhibitors to reduce the activity of PDE‐5, thereby reducing the hydrolysis of c‐GMP by PDE‐5 (Huang et al., 2023). It is worth noting that the product PC intervention effectively increased the content of cGMP in the mouse penile sponge. It can be seen that PC is rich in active substances in OP and PGE, which may effectively improve male ED caused by PRX by mediating NO/cGMP signaling pathway.

Continuous administration of PRX not only mediates the NO/cGMP signaling pathway to affect male sexual function, but also disrupts the synthesis and secretion of male sex hormones. Some previous studies have reported that PRX can lead to a significant decrease in serum hormone concentration in rats (Ajiboye et al., 2014). T plays an important role in promoting male gonadal development, maintaining male secondary sexual characteristics and spermatogenesis, and also affects male sexual desire, penile erectile function, and ejaculation function (Corona et al., 2022). Therefore, T deficiency can lead to male sexual dysfunction. In this study, the decrease of serum T content in male mice after PRX administration may be due to the toxic effect of the drug on the hypothalamic–pituitary–gonadal axis, or the interference of some enzyme activities in the process of T biosynthesis (Yakubu & Jimoh, 2015), resulting in a decrease in T concentration. The results of this study are similar to the results of previous studies (Ajiboye et al., 2014; Canpolat et al., 2022). Studies have shown that OP and *Pfaffia glomerata* have the effect of promoting T synthesis in male animals (Luo et al., 2021; Oshima & Gu, 2003). In this study, the product PC effectively reversed the low T level caused by PRX, indicating that the peptide or amino acid in OP and the triterpenoid saponins in PGE may be used as the precursor of T synthesis or stimulate T synthesis (El‐Shalofy et al., 2023; Yakubu & Jimoh, 2015), or increase the synthesis and secretion of T in Leydig cells by regulating the level of cGMP through the inhibition of PDE‐5 (Yadav & Mishra, 2024). The increase of LH level can also promote the production of T (Al‐Bader & Kilarkaje, 2015). Therefore, PC may effectively improve PRX‐induced sex hormone disorders in male mice by mediating the hypothalamic–pituitary–gonadal axis.

The imbalance between pro‐oxidation and anti‐oxidant systems can lead to oxidative stress damage in the male reproductive system, leading to male reproductive dysfunction (Barati et al., 2020). Ademosun et al. (2019) found that PRX could lead to oxidative damage in male rats, which was manifested by the increase of MDA content and the decrease of total thiol and nonprotein thiol levels. This phenomenon was also found in the study of Saikia et al. (2023), that is, PRX administration resulted in a decrease in antioxidant enzyme (SOD, CAT) activity and GSH content in testicular tissue of male mice, and an increase in MDA content. In this study, the SOD, CAT, and GSH‐Px activities of testicular tissue in the PRX group were significantly lower than those in the CN group, and the MDA content was significantly increased, which was similar to the previous results. The results of pathological sections of testicular tissue show that the morphological structure of testicular tissue in mice was destroyed after PRX administration, which may be due to the oxidative stress damage of PRX to testicular tissue in mice. However, after PC intervention, the activity of antioxidant enzymes was increased and the content of MDA was decreased, the morphological structure of testicular tissue was improved, and the content of serum sexual hormones was also significantly increased. It indicated that PC has strong antioxidant capacity and can alleviate oxidative stress damage caused by PRX, which may be due to the active substances such as active peptides, amino acid composition, and triterpenoid saponins in PC (Dias et al., 2019; Zhu et al., 2023).

In conclusion, PC has a complete range of amino acids, high proportions of EAA, HAA, and BCAA, and contains a certain amount of Zn and Se elements, and it has high nutritional value. At the same time, it is rich in triterpenoid saponins and other active substances. In addition, PC has a good ameliorative effect on PRX‐induced male sexual disorders and can be used as a dietary supplement to improve male sexual dysfunction. It is suggested that the synergistic compatibility of oyster peptides (animal resources) and plant extracts (plant resources) may be a potential effective combination to improve bioavailability. In addition, this study provides new inspiration for the deep processing and utilization of OP and PGE, as well as the development and application of OP‐ and PGE‐related functional products.

## AUTHOR CONTRIBUTIONS

**Qianqian Huang:** Conceptualization (equal); data curation (lead); formal analysis (lead); investigation (lead); methodology (lead); software (lead); validation (lead); visualization (lead); writing – original draft (lead); writing – review and editing (equal). **Haiying Wu:** Formal analysis (supporting); investigation (supporting); methodology (supporting); software (supporting); validation (supporting); visualization (supporting); writing – review and editing (supporting). **Xiangxin Xiao:** Formal analysis (supporting); investigation (supporting); methodology (supporting); software (supporting); validation (supporting); visualization (supporting); writing – review and editing (supporting). **Xiaoming Qin:** Conceptualization (equal); data curation (supporting); funding acquisition (lead); investigation (supporting); project administration (lead); resources (equal); supervision (lead); writing – original draft (supporting). **Suqing Liu:** Funding acquisition (equal); investigation (supporting); project administration (equal); resources (equal); supervision (lead); visualization (supporting); writing – review and editing (supporting).

## FUNDING INFORMATION

This research was financially supported by the China Agricultural Research Systems for Tilapia (CARS‐49) and a Study on introduction, cultivation and demonstration of Brazil ginseng under forest (2022KJCX002).

## CONFLICT OF INTEREST STATEMENT

The authors declare no conflict of interest.

## INSTITUTIONAL REVIEW BOARD STATEMENT

The whole animal experiment was approved by the Experimental Animal Committee of Guangdong Ocean University (no. GDOU‐LAE‐2022‐039).

## Data Availability

Data will be made available from the authors on request.
